# *Cryptoporus volvatus* Extract Inhibits Porcine Reproductive and Respiratory Syndrome Virus (PRRSV) *In Vitro* and *In Vivo*

**DOI:** 10.1371/journal.pone.0063767

**Published:** 2013-05-21

**Authors:** Li Gao, Weiwei Zhang, Yipeng Sun, Qian Yang, Jie Ren, Jinhua Liu, Hexiang Wang, Wen-hai Feng

**Affiliations:** 1 State Key Laboratory of Agrobiotechnology, China Agricultural University, Beijing, China; 2 Ministry of Agriculture Key Laboratory of Soil Microbiology, China Agricultural University, Beijing, China; 3 Department of Microbiology and Immunology, College of Biological Sciences, China Agricultural University, Beijing, China; 4 Key Laboratory of Animal Epidemiology and Zoonosis, Ministry of Agriculture, College of Veterinary Medicine, China Agricultural University, Beijing, China; University of Alabama at Birmingham, United States of America

## Abstract

Porcine reproductive and respiratory syndrome virus (PRRSV) is an important arterivirus that can cause significant losses in swine industry. At present, there are no adequate control strategies against PRRSV. Thus, there is an urgent need for new treatment regimens that have efficacious antiviral activity to compensate for vaccines. *Cryptoporus volvatus* commonly serves as an anti-infective agent in Tradational Chinese Medicines. In this report, we exploited whether the aqueous extract from the fruiting body of *Cryptoporus volvatus* had the potential to inhibit PRRSV infection. Our results showed that the extract significantly inhibited PRRSV infection by repressing virus entry, viral RNA expression, and possibly viral protein synthesis, cell-to-cell spread, and releasing of virus particles. However, it did not block PRRSV binding to cells. Further studies confirmed that the extract directly inhibited PRRSV RNA-dependent RNA polymerase (RdRp) activity, thus interfering with PRRSV RNA and protein synthesis. More importantly, the extract efficiently inhibited highly pathologic PRRSV (HP-PRRSV) infection *in vivo*, reduced virus load in serum, and increased the survival rate of pigs inoculated with HP-PRRSV strain. Collectively, our findings imply that the aqueous extract from the fruiting body of *Cryptoporus volvatus* has the potential to be used for anti-PRRSV therapies.

## Introduction

Porcine reproductive and respiratory syndrome (PRRS) is one of the most important diseases in the swine industry, causing significant economical losses worldwide [1], [2]. The causative agent, PRRS virus (PRRSV), can cause reproductive failures in pregnant sows, respiratory diseases in piglets, and asymptomatic infection in boars [1]. Most recently, there have been devastating outbreaks of atypical PRRS in China, which is characterized by high fever, high morbidity, and high mortality in pigs of all ages [3], [4]. The causative agent is a highly pathogenic PRRSV (HP-PRRSV) genotype with a discontinuous deletion of 30 amino acids in nonstructural protein 2 (nsp2) [3], [4], [5], even though it has been shown that this deletion has nothing to with its virulence [6]. PRRSV belongs to the family *Arteriviridae*, of the order *Nidovirales* [7], which also includes equine arteritis virus (EAV), simian hemorrhagic fever virus (SHFV), and lactate dehydrogenase-elevating virus (LDV) [8]. PRRSV genome is a single-stranded, positive-sense RNA, which is approximately 15.4 kb in length and contains two large open reading frames (ORF1a and 1b) and a set of eight downstream ORFs (ORF2a, 2b, 3, 4, 5a, 5, 6, and 7), encoding 8 structural proteins and 14 non-structural proteins [8], [9], [10]. Enzymes required for arterivirus RNA synthesis are encoded in ORF1b, in particular the viral RNA-dependent RNA polymerase (RdRp; nsp9) and RNA helicase (Hel; nsp10). Together with the putative “accessory subunits”, these enzymes assemble into a membrane-associated viral replication and transcription complex (RTC) [11], which mediates both genome replication and the synthesis of a nested set of subgenomic (sg) mRNAs.

Great efforts have been made to control and eradicate PRRSV infection since it was first reported in 1987. However, the currently commercialized vaccines including killed and modified live vaccines have not been successful in eradicating the virus and do not provide complete immunity from heterologous infections [12]. And also, there is a safety issue concerning about modified live vaccines, since there have been reports, showing that the virus shedding from the vaccinated animals often reverts to virulent strains [12], [13]. Antiviral therapeutics is a critical tool for combating virus infections, especially for virus that does not have vaccines to match well with the circulating virus. Thus, an alternative measure to control PRRSV is the pharmacological intervention. Previous studies have discovered a few natural compounds and compositions that have antivral activities on PRRSV [14]. However, until now there are no effective commercial drugs available to control PRRSV infection.

The medical use of mushrooms has a long tradition in Asian countries, and their use in the Western hemisphere has been slightly increased in the past decades [15], [16], [17], [18]. Antiviral effects are described not only for the whole extracts of mushrooms [19] but also for isolated compounds [20], [21]. *Cryptoporus volvatus* belongs to *Aphyllophorales, Cryptoporus* [22], and grows in certain areas in China. Its fruiting body was used for the treatment of asthma and bronchitis back to the 15^th^ century a.d. when the record of *Cryptoporus volvatus* appeared in “Materia Medica of Yunnan” [23]. Chemical analysis of *Cryptoporus volvatus* revealed that it contained many physiological activators, such as polysaccharose, proteins, volatile oil, and cryptoporic acids, etc [24]. Aqueous extract from the fruiting body of *Cryptoporus volvatus* has been reported to have anti-tumor, anti-allergy, anti-inflammation, and immunomodulatory activities [25], [26], [27]. However, there are no reports about its antiviral activity.

In the present study, we investigated whether the aqueous extract from the fruiting body of *Cryptoporus volvatus* had the ability to inhibit PRRSV infection. We first examined its potential to inhibit PRRSV replication *in vitro* and determined the stages in the PRRSV life cycle that could be blocked by the extract, and then extended our study in animal models to see if the extract could inhibit PRRSV infection *in vivo*. Our results showed that the extract from *Cryptoporus volvatus* inhibited PRRSV infection both *in vitro* and *in vivo*, implicating that the aqueous extract from the fruiting body of *Cryptoporus volvatus* has the potential to be used as an antiviral therapeutics.

## Materials and Methods

### Ethics Statement

All animal research was approved by the Beijing Association for Science and Technology (approval ID SYXK (Beijing) 2007–0023) and complied with the guidelines of Beijing Laboratory Animal Welfare and Ethics of the Beijing Administration Committee of Laboratory Animals. All animal studies were also performed in accordance with the China Agricultural University Institutional Animal Care and Use Committee guidelines (ID: SKLAB-B-2010-003) and approved by animal welfare committee of China Agricultural University. All surgery was performed under sodium pentobarbital anesthesia, and all efforts were made to minimize suffering.

### Cells, Viruses, and Virus Preparations

Marc-145 cells are a PRRSV-permissive cell line sub-cloned from MA-104 cells [28]. Marc-145 cells were maintained in Dulbecco’s minimum essential medium (DMEM) supplemented with 10% FBS and penicillin/streptomycin. Porcine alveolar macrophages (PAMs) were obtained by postmortem lung lavage of 8-week-old specific pathogen free (SPF) pigs, and maintained in RPMI 1640 supplemented with 10% FBS and penicillin/streptomycin.

PRRSV strains, CH-1a (the first type 2 PRRSV strain isolated in China), VR2332 (the prototype of Type 2 PRRSV strain), and HV (a highly pathogenic PRRSV (HP-PRRSV) isolate, GenBank accession no. JX317648), were propagated in Marc-145 cells or PAMs. Virus preparations were titrated on Marc-145 cells or PAMs, and then stored at −80°C. Briefly, PRRSV was serially diluted 10-fold in complete DMEM or RPMI1640 to infect 5×10^4^ Marc-145 cells or PAMs in 96-well plates. PRRSV infection was determined 72 h post infection using immunofluorescent staining for the PRRSV N protein. Virus titer was determined using Reed-Muench method, and expressed as tissue culture infective dose 50% (TCID_50_). PFU was determined according to “PFU = 0.7* TCID_50_” as described before [29], and the multiplicity of infection (MOI) was calculated based on PFU.

### Indirect Immunofluorescent Assay

Cells were fixed with cold methanol-acetone (1∶1) for 10 min at 4°C, washed with phosphate-buffered saline (PBS), and then blocked with 5% normal goat serum for 30 min at room temperature. After blocking, cells were stained with anti-PRRSV N protein monoclonal antibody SDOW17 (1∶10,000; Rural Technologies), or an isotype control antibody for 60 min at room temperature. Cells were then washed and incubated with FITC-conjugated goat anti-mouse IgG (H+L) (1∶2000, Jackson ImmunoResearch) for 60 min at 37°C. After three washes in PBS, cells were counter-stained with DAPI and examined by fluorescence microscopy.

### Preparation of the *Cryptoporus volvatus* Extract

The dry fruiting body of the *Cryptoporus volvatus* was crushed and soaked in water overnight at 4°C, and then centrifuged at 8000–10000 g for 30 min. The supernatant was harvested and freeze-dried, and then stored at −80°C until use. When used, the freeze-dried powder was re-dissolved with normal saline or culture medium and filtered with 0.22 µm filters.

### MTT Assay

The MTT [3-(4,5-dimethyl-2-thiazolyl)-2,5-diphenyl-2H-tetrazo-lium bromide] assay was used to examine the effect of the *Cryptoporus volvatus* extract on cell viability. Marc-145 cells or PAMs in 96-well plates were treated with sequential dilutions of the extract or normal saline in a total of 100 µl growth medium for 48 h. And then, 20 µl of freshly made 5 mg/ml MTT solution was added to each well, and the cells were incubated at 37°C for another 5 h before the medium was replaced with 200 µl DMSO to dissolve the crystals. The plates were further incubated at 37°C for 5 min to dissolve any air bubbles before the MTT signal was measured at an absorbance of 550 nm. The 50% cytotoxic concentration (CC_50_) was analyzed by GraphPad Prism (GraphPad Software, San Diego, CA).

### Inhibition of Virus Infection Assay

Marc-145 cells or PAMs in 96-well plates were inoculated with Ch-1a or HV at an MOI of 0.1 for 2 h at 37°C, and then the viral inoculum was removed and fresh medium containing 2% FBS and different concentrations of the *Cryptoporus volvatus* extract or IFN-α (10 units/µl, kindly provided by Dr. Wenjun Liu, Chinese Academy of Sciences, Beijing, China), a known inhibitor of PRRSV replication [30], was added. Twenty-four hours later, the supernatant was collected for virus titration, and cells were fixed for indirect immunofluorescent assay. The 50% effective concentration (EC_50_) was determined using a 4 parameter, nonlinear regression of dose response inhibition by plotting log (inhibitor(concentration)) vs. virus titer (variable slope) using GraphPad Prism (GraphPad Software, San Diego, CA).

### Virus Attachment and Entry Assay

For attachment assay, Marc-145 cells were inoculated with Ch-1a (MOI = 1) at 4°C for 2 h in the presence or absence of different concentrations of the *Cryptoporus volvatus* extract, and then cell lysates were prepared by freeze-thaw three times after cells were washed 3 times with cold PBS. Virus titer was determined as described above. For entry assay, Marc-145 cells were incubated with Ch-1a (MOI = 1) at 4°C for 2 h. Next, the viral inoculum was removed and cells were washed 3 times with cold PBS to remove unattached virus particles. Then, fresh medium was added and cell cultures were switched to 37°C (this time point was set up as 0 h). Cell culture medium was replaced with fresh medium containing different concentrations of the *Cryptoporus volvatus* extract at 0, 1, 2, or 3 h following temperature switch. At 5 h following temperature switch, medium was replaced with fresh medium, and cells were further incubated at 37°C. Twenty-four hours later, supernatants were harvested for virus titration.

For entry assay, confocal microscopy was also performed. Briefly, Marc-145 cells were incubated with Ch-1a (MOI = 50) at 4°C for 2 h. And then, cells were fixed with cold methanol-acetone after 3 washes with cold PBS or continued to be cultured in fresh medium with or without the *Cryptoporus volvatus* extract at 37°C for another 3 hours before being fixed with cold methanol-acetone. Fixed cells were stained for PRRSV N protein as described above using IFA and labeled F-actin with Phalloidin -TRITC (Sigma) following manufacturer’s protocol. Immunofluorescence was observed using Leica Microsystems CMS GmbH.

### RNA Extraction and Real-time PCR

Total RNA was extracted using Trizol Reagent (Invitrogen) and 1 µg RNA was used for cDNA synthesis using M-MLV (Promega). Real-time PCR was performed using specific primers for PRRSV N protein gene (forward: AATAACAACGGCAAGCAGCA, Reverse: GCACAGTATGATGCGTCGGC) by ABI7500 Real Time PCR System using the Real Time SYBR master mix kit (TAKARA) following the manufacturer’s introductions. For each experiment, a standard curve was generated using serially diluted PRRSV standard of 10^0^–10^7^ TCID_50_/ml.

### Western Blot Analysis

Whole-cell extracts were prepared with RIPA lysis buffer supplemented with protease inhibitors. Same amount of proteins from each sample was separated by 12% sodium dodecyl sulfate-polyacrylamide gel electrophoresis (SDS-PAGE) and transferred to PVDF membranes. After blocking, the membranes were incubated for 1 h at room temperature with the following primary antibodies diluted as indicated: anti-PRRSV N monoclonal antibody protein SDOW17 (1∶5,000; Rural Technologies), and anti-β-actin antibody (1∶5,000; Sigma, St.Louis, MO). The membranes were then incubated with the appropriate secondary antibody for 1 hour (1∶5,000). The antibodies were visualized by use of the ECL reagent according to the manufacturer’s instructions.

### Expression and Purification of the PRRSV nsp9/RdRp (7474–9526)

The cDNA sequence encoding the PRRSV nsp9**/**RdRp (7474–9526) was the reverse transcription product of the high-pathogenicity PRRSV strain HV. The ORF1a/ORF1b ribosomal frameshift site was removed by mutating nucleotides T-7600 and A-7602 to C, and inserting a C between A-7604 and C-7605 [31], [32], which were showed in lowercase in the primers. Nsp9 (7474–7614) and nsp9 (7593–9526) segments were individually amplified using primers nsp9 (7474–7614) forward: 5′-GTTGGCATATGGCCGCCAAGCTTTCC-3′, nsp9 (7474–7614) reverse: 5′-GGCTAGCAgGTTgAgACACTGCTCCTTAGTCAGG-3′; nsp9 (7593–9526) forward: 5′-GCAGTGTcTcAAcCTGCTAGCCGCCAGC-3′, nsp9 (7593–9526) reverse: 5′-CCGGATCCTTACTCATGATTGGACCTGAGTTTTTCC-3′). And then, nsp9 (7474–9526) cDNA was obtained by overlap-extension PCR of the sequences of nsp9 (7474–7614) and nsp9 (7593–9526). The nsp9/RdRp (7474–9526) was then inserted between the Nde I and Bam HI restriction sites of pET28 expression vector (Novagen) and sequenced, giving rise to construct pET28-nsp9/RdRpNHis.

The protein was expressed in *E. coli* BL21-CodonPlus (DE3)-RIL cells after overnight induction with 1 mM IPTG at 25°C. The bacterial pellet was re-suspended in lysis buffer (50 mM Tris [pH 7.4], 10 mM imidazole [pH 7.4], 500 mM NaCl, 2.5 mM MgCl_2_, 0.5 mg/ml lysozyme, 1 mM PMSF and 0.1 mg/ml DNase I), incubated for 30 min at room temperature, and finally sonicated. The protein was purified from the soluble fraction of the bacterial lysate by loading onto a nickel-nitrilotriacetic acid (Ni-NTA) column. The protein was eluted with 250 mM imidazole, and then concentrated using Amicon Ultra-15 Centrifugal Filter Units (Millipore). The purified protein was preserved in storage buffer (20 mM Tris [pH 7.4], 100 mM NaCl, 1 mM dithiothreitol [DTT], 0.1 mM EDTA, 10% glycerol), and kept at −80°C. The yield was approximately 16 mg/liter of *E. coli* culture.

### Polymerase Activity Assay

Homopolymeric template poly(U)_18_ was synthesized by Shanghai Genepharma Co. Ltd. (China). To analyze the effect of the *Cryptoporus volvatus* extract on RNA-dependent RNA polymerase (RdRp) activity, we used filter-binding assays. Reactions were performed as previously described [32] in 50 µl of RdRp buffer (50 mM HEPES [pH 8.0], 10 mM KCl, 1 mM DTT, 2 mM MnCl_2_, and 4 mM MgCl_2_) containing 400 nM poly(U)_18_ template, 2 µM PRRSV nsp9/RdRp, 0.5 µCi [^32^P]ATP, ATP 100 µm,RNase inhibitor 50 units, and the *Cryptoporus volvatus* extract with final concentrations of 0, 0.05, 0.25, and 0.5 mg/ml. IFN-α and Cordycepin (Shanghai Tongjibiological, China) were used as controls, and the final concentrations of IFN-α and Cordycepin were 10 units/µl and 1 mM, respectively [33], [34]. The reaction was performed at 30°C for 1 h, and stopped by addition of an equal volume of 50 mM EDTA. And then, the mixture was spotted onto DE-81 filter paper (Whatman). The filters were washed three times with 0.3 M ammonium formate (pH 8.0) and once with ethanol. After filters were dried, liquid scintillation fluid was added. Incorporation was then measured in counts per minute (cpm) using a Wallac Micro- Beta liquid scintillation counter.

### Virus Release Assay

Marc-145 cells were infected with Ch-1a (MOI = 0.1). At 24 h post infection (h.p.i), cells were washed 3 times with PBS and replaced with fresh medium containing different concentrations of the *Cryptoporus volvatus* extract or BFA (1 µg/ml). At 0.5 h, 1 h, and 3 h following medium replacement, supernatants were collected and cells were lysed by rapid freeze-thaw on dry ice-ethanol and a 37°C water bath. PRRSV RNA copies in the supernatants (extracellular virus) and cell lysates (intracellular virus) were then quantified using quantitative real-time PCR.

### PRRSV Cell-to-cell Transmission Assay

Confluent monolayers of Marc-145 cells were inoculated with Ch-1a (MOI = 0.01) at 37°C for 3 h, and then fresh medium containing neutralizing antibody and the *Cryptoporus volvatus* extract at different final concentrations was added after the cells were washed three times with PBS. Cells were further incubated at 37°C for 24 h, and then detected for PRRSV N expression by indirect immunofluorescence. The mean number of infected cells/foci was determined from ≥100 foci for each data point.

### Treatment Study in Piglets

Ten 4-week-old SPF Landrace piglets were obtained from the Beijing Center for SPF Swine Breeding and Management. The animals were randomly assigned to two groups (five piglets per group). Each group was housed separately in a different isolation room with individual ventilation. Each piglet was intranasally inoculated with 2 ml of HP-PRRSV strain HV containing 10^5.0^ TCID_50_ virus/ml. The piglets in the treatment group were administered intraperitoneally and intramuscularly with the *Cryptoporus volvatus* extract twice daily (7.5 mg extract/kg body weight each time, half for i.p., and the other half for i.m.) for 8 days. The piglets in the control group were mock administered with the same volume of normal saline. The animals were observed daily for clinical signs, and rectal temperatures were measured every day for 20 days post inoculation (dpi). Serum was collected at 3, 7, 10, 16 and 45 dpi for the detection of viral RNA by quantitative RT-PCR.

### Statistical Analysis

All experiments were performed with at least three independent replicates. Results were analyzed using *Student’s t test*. Differences were considered to be statistically significant if the *P* value is less than 0.05. *P<0.05; **P<0.01; ***P<0.001.

## Results

### *Cryptoporus volvatus* Extract Inhibits PRRSV Replication

**Figure 1:**
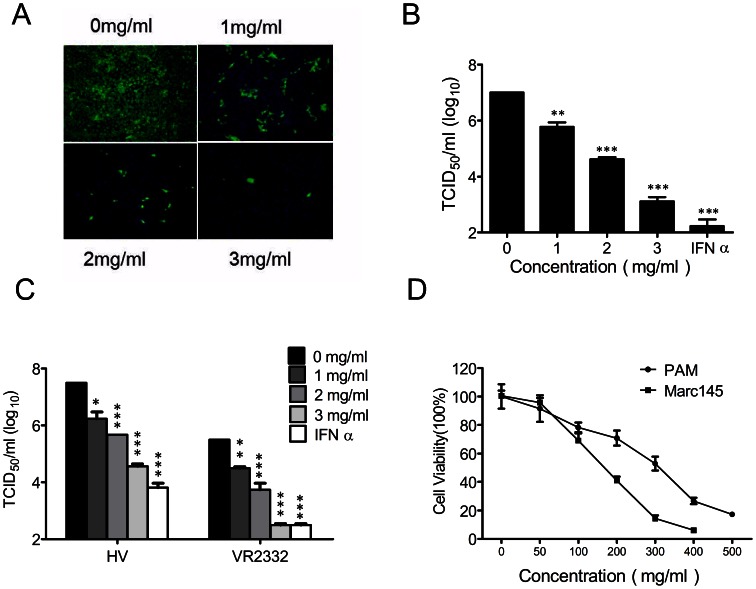
*Cryptoporus volvatus* extract inhibits PRRSV replication. (A and B) The *Cryptoporus volvatus* extract blocks PRRSV Ch1a replication in Marc-145 cells. Marc-145 cells were infected with PRRSV Ch1a at an MOI of 0.1, and then treated with IFN-α (10 units/µl) or the *Cryptoporus volvatus* extract at various concentrations. At 24 h p.i., cells were fixed and analyzed by IFA using antibody against PRRSV N protein (A), and virus yield in the supernatants was also quantified (B). Cultures treated with normal saline were set up as control (0 mg/ml). Results are from three independent experiments, each of which was in triplicate. (C) *Cryptoporus volvatus* extract potently inhibits both PRRSV VR2332 and HV replication in PAMs. A similar virus inhibition assay was performed with PAM cells infected with PRRSV strain VR2332 or HV at an MOI of 0.1 in the presence of IFN-α (10 units/µl) or the *Cryptoporus volvatus* extract at various concentrations. (D) Determination of cytotoxicity of the *Cryptoporus volvatus* extract by MTT assay. PAMs or Marc145 cells were incubated with various concentrations of the *Cryptoporus volvatus* extract or the control normal saline for 48 h prior to the MTT assay. Data are representative of three independent experiments (mean ± SD). Statistical significance was analyzed by *Student’s t test*. *P<0.05; **P<0.01; ***P<0.001.

To explore the antiviral activity of the *Cryptoporus volvatus* extract against viruses, we first investigated its antiviral effect on PRRSV infection. As shown in Fig. 1A and B, the *Cryptoporus volvatus* extract significantly inhibited PRRSV (CH-1a strain) replication in Marc-145 cells. The extract reduced the virus yields about 10^4^-fold at the concentration of 3 mg/ml when compared to normal saline control, and this inhibition was in a dose-dependent manner. To further verify its anti-PRRSV activity, we examined whether the *Cryptoporus volvatus* extract could inhibit replication of more than one PRRSV strain in PAMs. As illustrated in Fig. 1C, the *Cryptoporus volvatus* extract potently inhibited both the prototype of Type 2 PRRSV strain (VR2332) and HP-PRRSV strain (HV) replications in PAMs, which could reach a 1000-fold suppression at the concentration of 3 mg/ml. The extract inhibited PRRSV infection with 50% effective concentration (EC_50_) values of 0.34 mg/ml for CH-1a strain in Marc-145 cells, 0.34 mg/ml for HV strain and 0.36 mg/ml for VR2332 in PAMs.

To exclude the possibility that nonspecific toxicity induced by the extract could affect PRRSV replication, we evaluated PAM and Marc-145 cell viability under various concentrations of the *Cryptoporus volvatus* extract using the MTT assay (Fig. 1D). The 50% cytotoxic concentrations (CC_50_) of the *Cryptoporus volvatus* extract for PAMs and Marc-145 cells were 263 mg/ml and 147 mg/ml, respectively, which greatly exceeded its EC_50_. The therapeutic index (CC_50_/EC_50_) was 600 for CH-1a strain in Marc-145 cells, 947 for HV strain and 894 for VR2332 in PAMs.

These initial studies confirmed that the *Cryptoporus volvatus* extract could inhibit PRRSV infection. Therefore, we characterized the specific step(s) of the PRRSV life cycle that could be impeded by the extract in subsequent works.

### *Cryptoporus volvatus* Extract Inhibits PRRSV Entry but not Attachment

**Figure 2:**
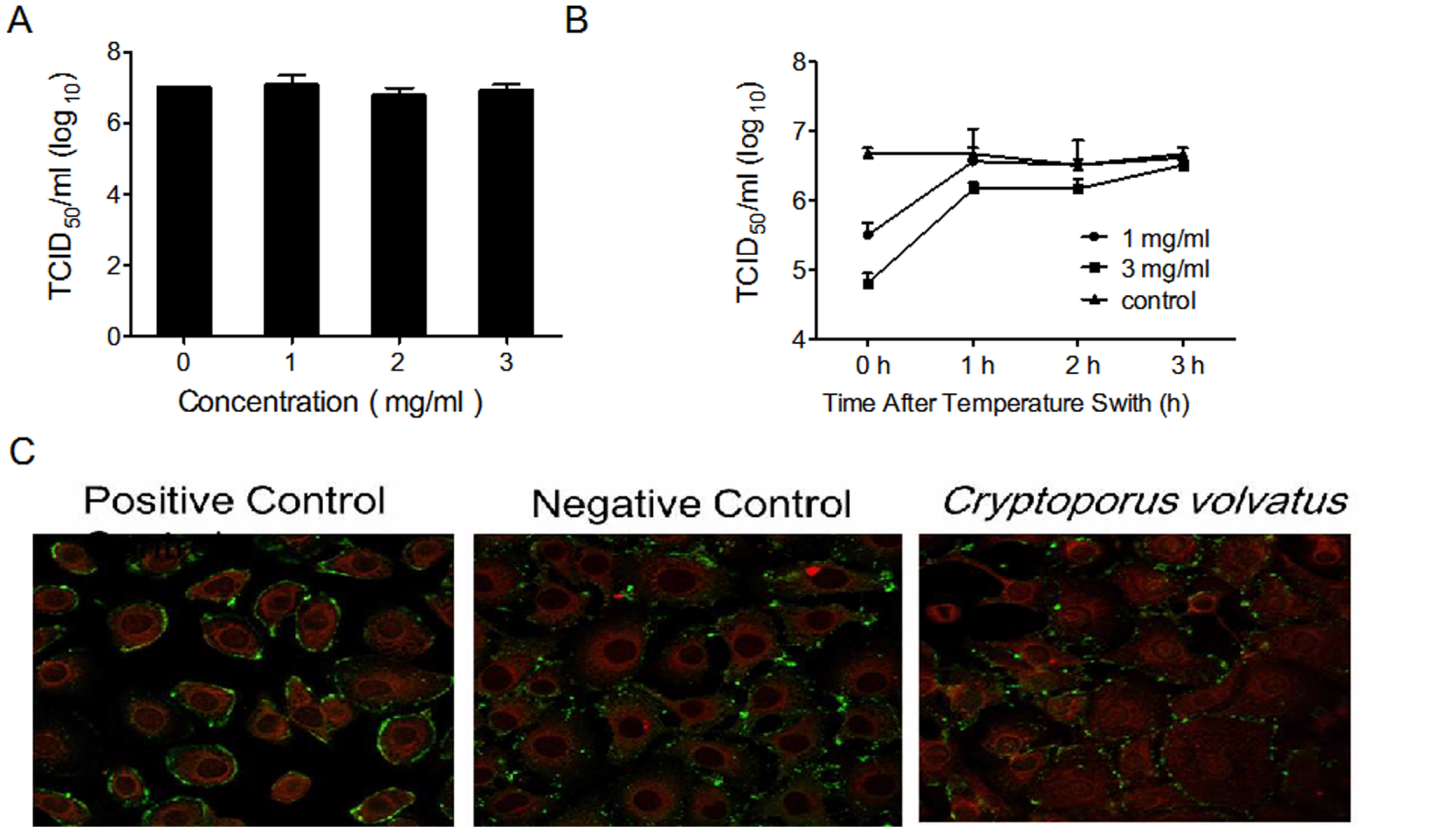
*Cryptoporus volvatus* extract inhibits PRRSV entry into Marc-145 cell but not attachment. (A) Effects of the *Cryptoporus volvatus* extract on virus attachment. Marc-145 cells were inoculated with Ch1a (MOI = 1) at 4°C for 2 h with different concentrations of the extract, and then cell lysates were prepared by freeze-thaw three times after cells were washed 3 times with cold PBS. Virus titer (TCID_50_) was determined. (B) Inhibition of PRRSV entry by the *Cryptoporus volvatus* extract. Marc-145 cells were incubated with Ch1a (MOI = 1) at 4°C for 2 h. Unbound virus was removed by washing three times with cold PBS, and the temperature was switched to 37°C (this time point was set up as 0 h). Cell medium was replaced with fresh medium containing different concentrations of the extract at 0, 1, 2, or 3 h following temperature switch. Five hours after temperature switch, medium was replaced with fresh medium, and cells were further incubated at 37°C. Twenty-four hours later, supernatants were harvested for virus titration. (C) Confocal analysis showing that *Cryptoporus volvatus* extract inhibits PRRSV entry into Marc145 cells. Marc-145 cells were incubated with Ch-1a (MOI = 50) at 4°C for 2 h. And then, cells were fixed with cold methanol-acetone after 3 washes with cold PBS (negative control) or continued to be cultured in fresh medium with or without (positive control) the *Cryptoporus volvatus* extract at 37°C for another 3 hours before being fixed with cold methanol-acetone after 3 washes with cold PBS. Fixed cells were stained for PRRSV N protein and labeled with Phalloidin -TRITC (Sigma). Immunofluorescence was observed using Leica Microsystems CMS GmbH. Data are representative of three independent experiments (mean ± SD). Statistical significance was analyzed using *Student’s t test*.

The process of PRRSV entry includes early attachment and internalization. To determine whether the *Cryptoporus volvatus* extract could inhibit PRRSV attachment on Marc-145 cells, virus-cell binding assay was performed at 4°C under conditions in which virus binds to but does not enter cells. Our results demonstrated that the extract had no effects on virus replication when it was only present during the virus binding, suggesting that the *Cryptoporus volvatus* extract did not affect the quantity of infectious virus particles that attached to the cell membranes (Fig. 2A).

Previous studies have reported that PRRSV is internalized from the surface of Marc-145 cells within 3–6 hours [35], [36]. Thus, to test whether the *Cryptoporus volvatus* extract acts at the internalization stage of infection, we examined the kinetics of the extract activity against PRRSV infection using time-of-addition assays (Fig. 2B). The *Cryptoporus volvatus* extract caused approximately a 15-fold decrease of virus titers in cells infected with CH-1a at an MOI of 1 when the extract (1 mg/ml) was added right after temperature switch (present in the first 5 hours of the virus internalization process), and more significant reduction (∼80-fold decrease) was observed when the extract was used at the concentration of 3 mg/ml. When the extract (3 mg/ml) was added at 1 or 2 h after the culture was switched to 37°C, it only induced a 3.1-, or 2.1-fold suppression of virus replication compared to the control. However, no inhibition was observed when the extract was added 3–5 h following temperature switch. These data demonstrated that the inhibition of PRRSV internalization by the extract was exerted within the first 3 hours of infection. To further verify that the *Cryptoporus volvatus* extract could inhibit PRRSV internalization, we performed confocal microscopic analysis of PRRSV attachment and internalization in Marc-145 cells. As shown in Fig. 2C, internalized PRRSV particles were clearly stained and smoothly distributed inside of the cell membranes without extract-treatment (positive control), while most of the virus particles were clustered outside of the cells treated with the *Cryptoporus volvatus* extract, which was similar to the staining of the negative control (cells were fixed before virus internalization and then stained for PRRSV N protein and F-actin).

Taken together, these data showed that the *Cryptoporus volvatus* extract could inhibit PRRSV internalization but not attachment to the host cells.

### *Cryptoporus volvatus* Extract Strongly Inhibits PRRSV RNA and Protein Synthesis

**Figure 3:**
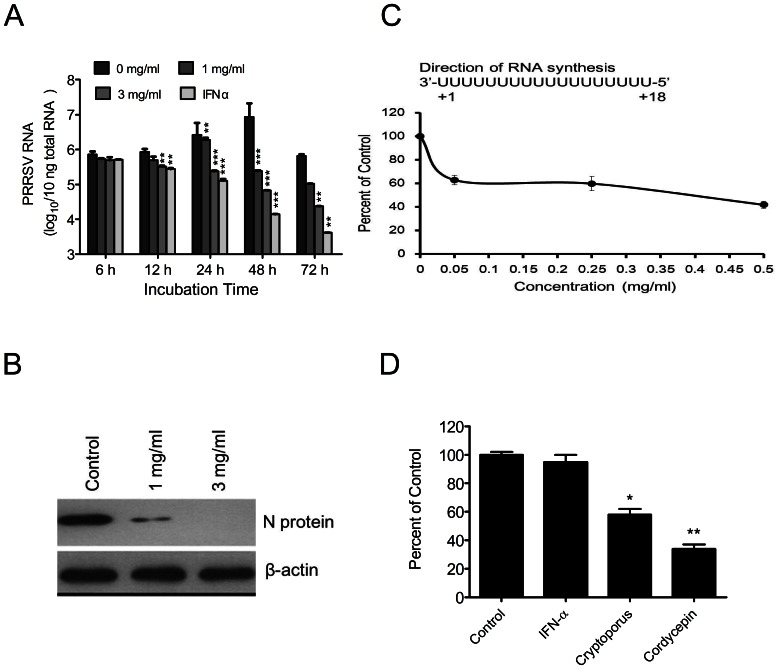
PRRSV RNA and protein synthesis are strongly inhibited by *Cryptoporus volvatus* extract. (A) *Cryptoporus volvatus* extract inhibits PRRSV RNA synthesis. Marc-145 cells were infected with PRRSV Ch1a at an MOI of 0.01. Twenty-four hours post infection, cells were then treated with various concentrations of the extract or IFN-α (10 units/µl). Whole-cell RNA was isolated from the cells at 6, 12, 24, 48, or 72 hours after treatment and analyzed for PRRSV RNA using a quantitative real-time RT-PCR assay. (B) PRRSV protein synthesis is inhibited by *Cryptoporus volvatus* extract. Marc-145 cells were infected with PRRSV Ch1a (MOI = 5) and then treated with *Cryptoporus volvatus* extract at 1 mg/ml or 3 mg/ml. Thirty-six hours later, whole-cell extracts were prepared for western blot analysis, and PRRSV protein was analyzed using anti-PRRSV N monoclonal antibody (SDOW17). (C) *Cryptoporus volvatus* extract directly inhibits the PRRSV RNA dependent RNA polymerase activity. PRRSV nsp9/RdRp was incubated with *Cryptoporus volvatus* extract at a final concentration of 0, 0.05, 0.25, or 0.5 mg/ml, and the activity of the PRRSV nsp9/RdRp was examined using filter-binding assays to monitor incorporation of [^32^P]ATP by using a poly(U)_18_ RNA template. (D) Cordycepin inhibits PRRSV RdRp activity but IFN-α not. Similar experiment as C was done with IFN-α, *Cryptoporus volvatus* extract, and Cordycepin at final concentrations of 10 units/µl, 0.5 mg/ml, 1 mM, respectively. Data are representative of three independent experiments (mean ± SD). Statistical significance was analyzed by *Student’s t test*; *P<0.05; **P<0.01; ***P<0.001.

Next, we examined whether the *Cryptoporus volvatus* extract could inhibit viral RNA synthesis in virus-infected cells. In this experiment, we first infected Marc-145 cells with CH-1a at an MOI of 0.01 for 24 hours, and then treated with the extract, or IFN-α (10 units/µl) (as positive control). The intracellular PRRSV RNA was measured by quantitative real-time PCR at different time points following treatment. As shown in Fig. 3A, relative to untreated cells, the extract caused a significant reduction in PRRSV RNA production at 12, 24, 48, and 72 hours after treatment. And the most significant suppression of PRRSV RNA production was observed at 48 hours after treatment, and the copies of viral RNA were decreased more than 140- folds. Consequently, PRRSV N protein expression was severely impaired by the extract (Fig. 3B).

We next examined whether the *Cryptoporus volvatus* extract could directly inhibit PRRSV RNA dependent RNA polymerase (RdRp) activity. PRRSV nsp9/RdRp was cloned and expressed [32], and the inhibition of the extract on the activity of the purified PRRSV nsp9/RdRp was examined using filter-binding assays to monitor incorporation of [^32^P]ATP by using a poly(U)_ 18_ RNA as template. As shown in Fig. 3C, the extract significantly inhibited the activity of the PRRSV nsp9/RdRp, with a 40% inhibition of the RdRp activity relative to the control at the concentration of 0.05 mg/ml. As expected, Cordycepin, which is an RNA polymerase inhibitor, also inhibited the activity of the PRRSV nsp9/RdRp (Fig. 3D). These results indicated that the extract could effectively inhibit the RdRp activity.

### *Cryptoporus volvatus* Extract Inhibits the Release of PRRSV Particles

**Figure 4:**
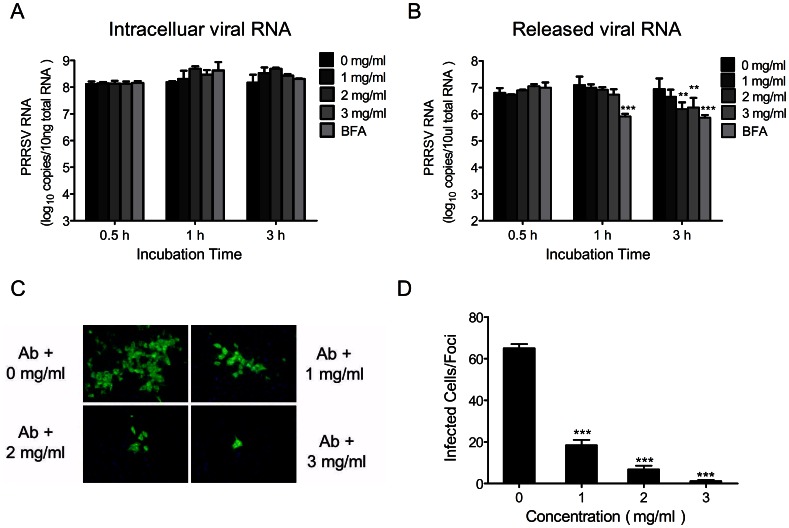
*Cryptoporus volvatus* extract inhibits PRRSV release and cell-to-cell spreading. Determination of viral RNA released to the supernatants (A) or intracellular (B). Marc-145 cells were infected with PRRSV Ch1a (MOI of 0.1) for 24 h. After washing with PBS, cells were treated with either *Cryptoporus volvatus* extract or BFA (a known inhibitor of protein transport) for 0.5, 1, or 3 hours, and then copies of viral RNA in the supernatants and in the cells were determined using quantitative real-time RT-PCR assay. (C) PRRSV cell-to-cell spread is inhibited by *Cryptoporus volvatus* extract in a dose-dependent manner. Marc145-cells were incubated with CH-1a strain (MOI = 0.01) for 3 h at 37°C, and then culture medium was replaced with fresh medium containing *Cryptoporus volvatus* extract at indicated concentrations and neutralizing antibody (10% vol/vol). PRRSV N protein expression in cells was observed using indirect immunofluorescence at 24 hours after infection. Micrographs chosen to illustrate the sizes of infected cell foci (N expression, green). (D) Average number of infected cells per foci. Data are representative of three independent experiments (mean ± SD). Statistical significance was analyzed by *Student’s t test*; *P<0.05; **P<0.01; ***P<0.001.

To determine whether the *Cryptoporus volvatus* extract affects virus release, we used an assay described previously [37] to quantify viruses that are either in cells or released into the supernatants. We first infected Marc-145 cells with CH-1a (MOI = 0.1). Twenty-four hours post infection, cells were extensively washed with PBS and then replaced with fresh medium containing different concentrations of the extract or BFA (1 µg/ml), a known inhibitor of protein transport [38]. Viral RNA copies that were either in cells or released into the supernatants were then quantified at 0.5 h, 1 h, and 3 h following treatments. At each time points, comparable amounts of intracellular viral RNAs were found in either extract or BFA-treated samples (Fig. 4A). In contrast, the copies of released viral RNA in supernatants significantly dropped by ∼80% when treated with the extract at the concentration of 2 mg/ml or 3 mg/ml for 3 h compared to the control (Fig. 4B). There was no significant effects observed when the extract was at 1 mg/ml. Our data suggested that the extract might block PRRSV virus particle release.

### *Cryptoporus volvatus* Extract Inhibits Direct Cell-to-cell Spread of PRRSV

The decrease of virus production observed in the extract-treated cultures could result from the production of fewer viruses in each infected cell or a failure of the virus to spread efficiently. There are two modes by which viruses can spread. Extracellular viruses are able to bind and enter an uninfected cell, or viruses inside cells can spread directly to adjacent cells without passing through a cell-free stage [39], [40]. PRRSV can spread in cultures of Marc-145 cells by both mechanisms [41]. To test the possibility that the *Cryptoporus volvatus* extract inhibits direct cell-to-cell spread of PRRSV, we monitored cell-to-cell virus spread in the presence of hyperimmune PRRSV-specific globulin. The hyperimmune globulin was titrated so that it neutralized as much infectious units of PRRSV as the amount produced in the infected culture cells in the absence of the extract. And in this condition, PRRSV could only spread by the way of cell-to-cell infection. Marc-145 cells were infected at an MOI of 0.0l, and globulin was added immediately thereafter. We observed a reduction of PRRSV-infected cells and the formation of discrete infected foci by addition of antibody in the absence of the extract using immunofluorescence assay compared to untreated controls (not shown here), suggesting that the cell-free virus spreading is prevented. When the extract was added, the size of the isolated PRRSV-infected foci was decreased in a dose-dependent manner (Fig. 4C). At twenty-four hours after infection, even the lowest concentration (1 mg/ml) of the extract tested remarkably reduced the size of infected foci (from ∼65 to 18 infected cells), and the average number of cells in an infected foci was only 1.25 when the extract was used at 3 mg/ml (Fig. 4D). Taken together, our results indicated that the *Cryptoporus volvatus* extract could inhibit PRRSV cell-to-cell spread.

### *Cryptoporus Volvatus* Extract Inhibits PRRSV Infection *in vivo*

**Figure 5:**
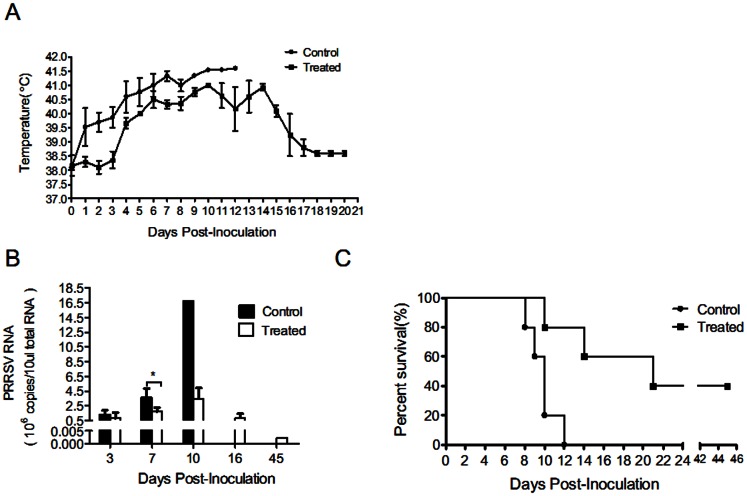
*Cryptoporus volvatus* extract inhibits PRRSV infection in vivo. (A) Rectal temperature measurements daily for each group of pigs(n = 5)after HP-PRRSV HV strain infection. (B) Analysis of viral loads in sera from each group of pigs. (C) Survive curves of pigs in each group. Data are presented as mean ± SE (A and B). Statistical significance was analyzed by *Student’s t test*; *P<0.05; **P<0.01; ***P<0.001.

To evaluate the inhibitory activity of the *Cryptoporus volvatus* extract *in vivo*, we tested its anti-PRRSV effects in HP-PRRSV strain HV-infected pigs. Four-week-old SPF piglets were intranasally challenged with HP-PRRSV strain HV (each with 2 ml of 10^5.0^ TCID_50_ PRRSV), and were then treated with the extract following virus challenge for 8 days (7.5 mg extract/kg body weight intraperitoneally (i.p.) and intramuscularly (i.m.)). It is well known that HP-PRRSV strain infection is characterized by high fever, high morbidity, and high mortality in pigs [2]. Therefore, we recorded the rectal temperature of each pig daily for 20 days or until it died (Fig. 5A). On day 4 post PRRSV inoculation, pigs in control group which did not receive the extract started to develop elevated body temperatures (>40°C) and remained higher than 40.5°C from day 4 post HV infection (Fig. 5A). Even though pigs in the treated group also had elevated body temperatures (Fig. 5A), the average temperatures were lower than that of control. Meanwhile, PRRSV RNA copies were analyzed in the serum samples at 3, 7, 10, 16, and 45 days pi (Fig. 5B). Viral loads in pigs from both control group and treatment group rose rapidly to 10^8^ (RNA copies/ml) at 3 dpi. However, viral growth in control group continued to grow rapidly and peaked at 10 dpi, while the viral growth in treated pigs was comparatively slower. The average viral RNA loads were significantly higher in no-treatment pigs than that in treated group (2.2-fold higher) at day 7. HP-PRRSV HV was highly virulent and caused 100% mortality when animals were challenged with 2 ml of 10^5^ TCID_50_ PRRSV (Fig. 5C). However, 2 of 5 piglets survived in the extract-treated group (Fig. 5C). Pigs infected with HV without treatment showed clinical signs including high fever, coughing, dyspnoea, anorexia, chemosis, shivering, lameness, and skin cyanopathy. When treated with the extract, pigs developed clinical signs much slower compared to untreated pigs. For those two who survived in the treatment group did not develop temperature above 40.5°C and had less severe clinical signs. These results provided direct evidence that the *Cryptoporus volvatus* extract could efficiently inhibit PRRSV replication *in vivo*.

## Discussion

Chinese herbal medicines are a unique source of medical complexity and diversity, and they have been exploited extensively by many in pursuit of new antiviral agents [42]. *Cryptoporus volvatus* has a long medical use history for treating asthma and bronchitis in China [23]. However, its antiviral activity has not been investigated. Here, we reported that the aqueous extract from the fruiting body of *Cryptoporus volvatus* had potent antiviral effects against PRRSV infection. Our data demonstrated that the extract inhibited multiple steps in the PRRSV life cycle: most notably entry and PRRSV RNA synthesis, and possibly cell-cell spread and viral particle secretion. And most importantly, it inhibited HP-PRRSV strain HV replication *in vivo*, reduced clinical severity, and increased pig survival rate.

The extract reduced intracellular PRRSV RNA levels and protein synthesis, and consequently, the production of progeny virus in infected cells. Central in the life cycle of plus-strand RNA viruses is the process of RNA-templated RNA synthesis, which is required to replicate and transcribe the viral genome. All positive-strand RNA viruses encode an RNA-dependent RNA polymerase that functions as the catalytic subunit for viral RNA synthesis. This biochemical activity is not present in mammalian cells. Therefore, viral RdRp is an important target for drug discovery, and many drugs could target viral RdRp to inhibit virus replication [43], [44]. Indeed, we cloned PRRSV RdRp and tested if the extract had effects on its activity. Our results indicated that the extract interfered with PRRSV RdRp activity, suggesting that the *Cryptoporus volvatus* extract has the potential to impair PRRSV replication by targeting PRRSV RdRp.

We also showed that the extract inhibited PRRSV protein synthesis, infectious PRRSV particle release from cells, and cell-to-cell spread. However, these could be the secondary effects due to the inhibition of PRRSV entry and RNA polymerase activity. Therefore, more work needs to be done to verify these observations in future.

More importantly, in the animal study, we found that the extract could repress high pathogenic PRRSV (HV) replication in pigs. The outbreaks of high pathogenic PRRS in China have caused great economic losses to the pig industry, and until now there have been no effective vaccines to confer protection against HP-PRRSV. The HP-PRRSV is extremely high pathogenic, which is characterized by high fever, high morbidity, and high mortality [2]. The HP-PRRSV strain (HV) used in this study could cause 100% death rate. When challenged with 2 ml of 10^5^ TCID_ 50_, 4-week-old pigs died in two weeks [45]. Our data here showed that the average viral loads in serum and the rectal temperatures in pigs of the treatment group were significantly lower. Surprisingly, two of the five pigs which were challenged with HV (2 ml of 10^5^ TCID_ 50_) survived after treatment with the extract. And 2 of the 3 pigs died in the treatment group survived longer (one on day 14, and one died on day 21) compared to the pigs challenged with HV but without treatment (all died within 12 days). These results suggest that the *Cryptoporus volvatus* extract has the potential to be used to treat PRRSV infected pigs. However, we need to indicate that even though two pigs survived, they grew much slower than pigs without infection. Obviously, more work remains for us to do. In future, we will expand our study using more pigs to investigate how the extract affects virus shedding/transmission, weight loss, and pathogenic changes, etc.

Aqueous extract from the fruiting body of *Cryptoporus volvatus* is a crude extract, which contains many components. The antiviral effects of the extract could result from the mixture of active compounds rather than from a single chemical entity. The efficacy of Traditional Chinese Medicine is a characteristic of a complex mixture of chemical compounds present in the various herbs. The concept of combinatorial medicines has been exemplified by the drug cocktail used in the treatment of acquired immunodeficiency syndrome [46]. However, in order to develop new generation of antiviral agents, it is necessary to isolate and purify the active compounds in the aqueous extract from the fruiting body of *Cryptoporus volvatus*. Actually, our preliminary data suggest that more than one compound from the extract can repress PRRSV replication. Obviously, more work remains for us to do to identify the molecules in the *Cryptoporus volvatus* extract.

In conclusion, our findings revealed that the aqueous extract of *Cryptoporus volvatus* exhibited antiviral activity against PRRSV infection and replication, implicating that it has the potential to be developed into a new generation of antiviral agent. Further studies are in progress to identify the molecules that are responsible for the inhibitions of virus replication.

## References

[1] Neumann EJ, Kliebenstein JB, Johnson CD, Mabry JW, Bush EJ, et al (2005) Assessment of the economic impact of porcine reproductive and respiratory syndrome on swine production in the United States. J Am Vet Med Assoc 227(3): 385–392. 16121604 10.2460/javma.2005.227.385

[2] Tian K, Yu X, Zhao T, Feng Y, Cao Z, et al (2007) Emergence of fatal PRRSV variants: unparalleled outbreaks of atypical PRRS in China and molecular dissection of the unique hallmark. PLoS One 2: e526. 17565379 10.1371/journal.pone.0000526 PMC1885284

[3] Li Y, Wang X, Bo K, Tang B, Yang B, et al (2007) Emergence of a highly pathogenic porcine reproductive and respiratory syndrome virus in the Mid-Eastern region of China. Vet J 174: 577–584. 17869553 10.1016/j.tvjl.2007.07.032

[4] Zhou YJ, Hao XF, Tian ZJ, Tong GZ, Yoo D, et al (2008) Highly virulent porcine reproductive and respiratory syndrome virus emerged in China. Transbound Emerg Dis 55: 152–164. 18405338 10.1111/j.1865-1682.2008.01020.x

[5] Ni J, Yang S, Bounlom D, Yu X, Zhou Z, et al (2012) Emergence and pathogenicity of highly pathogenic Porcine reproductive and respiratory syndrome virus in Vientiane, Lao People’s Democratic Republic. J Vet Diagn Invest 24: 349–354. 22379051 10.1177/1040638711434111

[6] Zhou L, Zhang J, Zeng J, Yin S, Li Y, et al (2009) The 30-amino-acid deletion in the Nsp2 of highly pathogenic porcine reproductive and respiratory syndrome virus emerging in China is not related to its virulence. J Virol 83: 5156–5167. 19244318 10.1128/JVI.02678-08 PMC2682102

[7] Gorbalenya AE, Enjuanes L, Ziebuhr J, Snijder EJ (2006) Nidovirales: evolving the largest RNA virus genome. Virus Res 117: 17–37. 16503362 10.1016/j.virusres.2006.01.017 PMC7114179

[8] Snijder EJ, Meulenberg JJ (1998) The molecular biology of arteriviruses. J Gen Virol 79 (Pt 5): 961–979. 10.1099/0022-1317-79-5-961 9603311

[9] Firth AE, Zevenhoven-Dobbe JC, Wills NM, Go YY, Balasuriya UB, et al (2011) Discovery of a small arterivirus gene that overlaps the GP5 coding sequence and is important for virus production. J Gen Virol 92: 1097–1106. 21307223 10.1099/vir.0.029264-0 PMC3139419

[10] Johnson CR, Griggs TF, Gnanandarajah J, Murtaugh MP (2011) Novel structural protein in porcine reproductive and respiratory syndrome virus encoded by an alternative ORF5 present in all arteriviruses. J Gen Virol 92: 1107–1116. 21307222 10.1099/vir.0.030213-0 PMC3139420

[11] Pedersen KW, van der Meer Y, Roos N, Snijder EJ (1999) Open reading frame 1a-encoded subunits of the arterivirus replicase induce endoplasmic reticulum-derived double-membrane vesicles which carry the viral replication complex. J Virol 73: 2016–2026. 9971782 10.1128/jvi.73.3.2016-2026.1999 PMC104444

[12] Murtaugh MP, Genzow M (2011) Immunological solutions for treatment and prevention of porcine reproductive and respiratory syndrome (PRRS). Vaccine 29: 8192–8204. 21925560 10.1016/j.vaccine.2011.09.013

[13] Storgaard T, Oleksiewicz M, Botner A (1999) Examination of the selective pressures on a live PRRS vaccine virus. Arch Virol 144: 2389–2401. 10664392 10.1007/s007050050652

[14] Karuppannan AK, Wu KX, Qiang J, Chu JJ, Kwang J (2012) Natural compounds inhibiting the replication of Porcine reproductive and respiratory syndrome virus. Antiviral Res 94: 188–194. 22487208 10.1016/j.antiviral.2012.03.008 PMC7114079

[15] Zjawiony JK (2004) Biologically active compounds from Aphyllophorales (polypore) fungi. J Nat Prod 67: 300–310. 14987072 10.1021/np030372w

[16] Stamets P (2006) Can mushrooms help save the world? Interview by Bonnie J. Horrigan. Explore (NY) 2: 152–161. 16781630 10.1016/j.explore.2005.12.011

[17] Wasser SP (2011) Current findings, future trends, and unsolved problems in studies of medicinal mushrooms. Appl Microbiol Biotechnol 89: 1323–1332. 21190105 10.1007/s00253-010-3067-4

[18] Lindequist U, Niedermeyer TH, Julich WD (2005) The pharmacological potential of mushrooms. Evid Based Complement Alternat Med 2: 285–299. 16136207 10.1093/ecam/neh107 PMC1193547

[19] Faccin LC, Benati F, Rincao VP, Mantovani MS, Soares SA, et al (2007) Antiviral activity of aqueous and ethanol extracts and of an isolated polysaccharide from Agaricus brasiliensis against poliovirus type 1. Lett Appl Microbiol 45: 24–28. 17594456 10.1111/j.1472-765X.2007.02153.x

[20] Mothana RA, Awadh Ali NA, Jansen R, Wegner U, Mentel R, et al (2003) Antiviral lanostanoid triterpenes from the fungus Ganoderma pfeifferi. Fitoterapia 74: 177–180. 12628419 10.1016/s0367-326x(02)00305-2

[21] Yamamoto KA, Galhardi LC, Rincao VP, Soares SD, Vieira IG, et al (2012) Antiherpetic activity of an Agaricus brasiliensis polysaccharide, its sulfated derivative and fractions. Int J Biol Macromol 52C: 9–13. 10.1016/j.ijbiomac.2012.09.029 23043759

[22] Xu J (1997) Chinese medicinal mycology. Beijing: publishing house of Peking Union Medical College and China Medical University. 836 p.

[23] Wu ZY (1990) Xin-Hua Compendium of Materia Medica. Shanghai: Shanghai Science and Technology Publishing House. 735 p.

[24] Wu JZ GJ, Huang NL (1999) et.al (1999) Fermentation Culture & Analysis of Compositions of Cryptoporus Volvatus (Peck) Schear. Journal of Fujian College of TCM 9(1): 33–36.

[25] Jin SH, Xie QM, Lin XX, Deng YM, Chen JQ (2003) [Effect of Cryptoporus volvatus (Peck) Schear on leukotriene production from polymorphonuclear leukocytes in rats]. Zhongguo Zhong Yao Za Zhi 28: 650–653. 15139113

[26] Yao HY, Zhang LH, Shen J, Shen HJ, Jia YL, et al (2011) Cyptoporus polysaccharide prevents lipopolysaccharide-induced acute lung injury associated with down-regulating Toll-like receptor 2 expression. J Ethnopharmacol 137: 1267–1274. 21875662 10.1016/j.jep.2011.07.058

[27] Xie QM, Deng JF, Deng YM, Shao CS, Zhang H, et al (2006) Effects of cryptoporus polysaccharide on rat allergic rhinitis associated with inhibiting eotaxin mRNA expression. J Ethnopharmacol 107: 424–430. 16765544 10.1016/j.jep.2006.03.040

[28] Kim HS, Kwang J, Yoon IJ, Joo HS, Frey ML (1993) Enhanced replication of porcine reproductive and respiratory syndrome (PRRS) virus in a homogeneous subpopulation of MA-104 cell line. Arch Virol 133: 477–483. 8257302 10.1007/BF01313785

[29] Quinting B, Robert B, Letellier C, Boxus M, Kerkhofs P, et al (2007) Development of a 1-step enzyme-linked immunosorbent assay for the rapid diagnosis of bovine respiratory syncytial virus in postmortem specimens. J Vet Diagn Invest 19: 238–243. 17459851 10.1177/104063870701900302

[30] Luo R, Fang L, Jin H, Jiang Y, Wang D, et al (2011) Antiviral activity of type I and type III interferons against porcine reproductive and respiratory syndrome virus (PRRSV). Antiviral Res 91: 99–101. 21569798 10.1016/j.antiviral.2011.04.017

[31] Wootton S, Yoo D, Rogan D (2000) Full-length sequence of a Canadian porcine reproductive and respiratory syndrome virus (PRRSV) isolate. Arch Virol 145: 2297–2323. 11205119 10.1007/s007050070022 PMC7086845

[32] Beerens N, Selisko B, Ricagno S, Imbert I, van der Zanden L, et al (2007) De novo initiation of RNA synthesis by the arterivirus RNA-dependent RNA polymerase. J Virol 81: 8384–8395. 17537850 10.1128/JVI.00564-07 PMC1951334

[33] Panicali DL, Nair CN (1978) Effect of cordycepin triphosphate on in vitro RNA synthesis by picornavirus polymerase complexes. J Virol 25: 124–128. 202731 10.1128/jvi.25.1.124-128.1978 PMC353908

[34] White JL, Dawson WO (1979) Effect of cordycepin triphosphate on in vitro RNA synthesis by plant viral replicases. J Virol 29: 811–814. 16789174 10.1128/jvi.29.2.811-814.1979 PMC353220

[35] Kreutz LC, Ackermann MR (1996) Porcine reproductive and respiratory syndrome virus enters cells through a low pH-dependent endocytic pathway. Virus Res 42: 137–147. 8806181 10.1016/0168-1702(96)01313-5

[36] Nauwynck HJ, Duan X, Favoreel HW, Van Oostveldt P, Pensaert MB (1999) Entry of porcine reproductive and respiratory syndrome virus into porcine alveolar macrophages via receptor-mediated endocytosis. J Gen Virol 80 (Pt 2): 297–305. 10.1099/0022-1317-80-2-297 10073688

[37] Kumar N, Liang Y, Parslow TG (2011) Receptor tyrosine kinase inhibitors block multiple steps of influenza a virus replication. J Virol 85: 2818–2827. 21209112 10.1128/JVI.01969-10 PMC3067926

[38] Misumi Y, Miki K, Takatsuki A, Tamura G, Ikehara Y (1986) Novel blockade by brefeldin A of intracellular transport of secretory proteins in cultured rat hepatocytes. J Biol Chem 261: 11398–11403. 2426273

[39] Sattentau Q (2008) Avoiding the void: cell-to-cell spread of human viruses. Nat Rev Microbiol 6: 815–826. 18923409 10.1038/nrmicro1972

[40] Sattentau QJ (2011) The direct passage of animal viruses between cells. Curr Opin Virol 1: 396–402. 22440841 10.1016/j.coviro.2011.09.004

[41] Cafruny WA, Duman RG, Wong GH, Said S, Ward-Demo P, et al (2006) Porcine reproductive and respiratory syndrome virus (PRRSV) infection spreads by cell-to-cell transfer in cultured MARC-145 cells, is dependent on an intact cytoskeleton, and is suppressed by drug-targeting of cell permissiveness to virus infection. Virol J 3: 90. 17081295 10.1186/1743-422X-3-90 PMC1635561

[42] Harvey AL (2008) Natural products in drug discovery. Drug Discov Today 13: 894–901. 18691670 10.1016/j.drudis.2008.07.004

[43] Tomei L, Altamura S, Bartholomew L, Bisbocci M, Bailey C, et al (2004) Characterization of the inhibition of hepatitis C virus RNA replication by nonnucleosides. J Virol 78: 938–946. 14694125 10.1128/JVI.78.2.938-946.2004 PMC368780

[44] Gastaminza P, Whitten-Bauer C, Chisari FV (2010) Unbiased probing of the entire hepatitis C virus life cycle identifies clinical compounds that target multiple aspects of the infection. Proc Natl Acad Sci U S A 107: 291–296. 19995961 10.1073/pnas.0912966107 PMC2806752

[45] Guo XK, Zhang Q, Gao L, Li N, Chen XX, et al. (14 Nov 2012) Additional expression of microRNA-181 inhibits Porcine Reproductive and Respiratory Syndrome Virus replication and its implications for controlling virus infection. J Virol: doi:10.1128/JVI.02386–02312. 10.1128/JVI.02386-12 PMC3554091 23152505

[46] Balzarini J, Pelemans H, Karlsson A, De Clerc QE, Kleim JP (1996) Concomitant combination therapy for HIV infection preferable over sequential therapy with 3TC and non-nucleoside reverse transcriptase inhibitors. Proc Natl Acad Sci U S A 93: 13152–13157. 8917560 10.1073/pnas.93.23.13152 PMC24062

